# Patterns of Immune Activation in HIV and Non HIV Subjects and Its Relation to Cardiovascular Disease Risk

**DOI:** 10.3389/fimmu.2021.647805

**Published:** 2021-07-05

**Authors:** Alinda G. Vos, Caitlin N. Dodd, Eveline M. Delemarre, Stefan Nierkens, Celicia Serenata, Diederick E. Grobbee, Kerstin Klipstein-Grobusch, W. D. Francois Venter

**Affiliations:** ^1^ Julius Global Health, Julius Center for Health Sciences and Primary Care, University Medical Center Utrecht, Utrecht University, Utrecht, Netherlands; ^2^ Ezintsha, Faculty of Health Sciences, University of the Witwatersrand, Johannesburg, South Africa; ^3^ Center for Translational Immunology (CTI), University Medical Center Utrecht, Utrecht University, Utrecht, Netherlands; ^4^ Division of Epidemiology and Biostatistics, School of Public Health, Faculty of Health Sciences, University of the Witwatersrand, Johannesburg, South Africa

**Keywords:** immune markers, immune patterns, HIV, CVD (cardio vascular disease), ART (antiretroviral therapy)

## Abstract

**Introduction:**

Insight into inflammation patterns is needed to understand the pathophysiology of HIV and related cardiovascular disease (CVD). We assessed patterns of inflammation related to HIV infection and CVD risk assessed with carotid intima media thickness (CIMT).

**Methods:**

A cross-sectional study was performed in Johannesburg, South Africa, including participants with HIV who were virally suppressed on anti-retroviral therapy (ART) as well as HIV-negative participants who were family members or friends to the HIV-positive participants. Information was collected on CVD risk factors and CIMT. Inflammation was measured with the Olink panel ‘inflammation’, allowing to simultaneously assess 92 inflammation markers. Differences in inflammation patterns between HIV-positive and HIV-negative participants were explored using a principal component analysis (PCA) and ANCOVA. The impact of differentiating immune markers, as identified by ANCOVA, on CIMT was assessed using linear regression while adjusting for classic CVD risk factors.

**Results:**

In total, 185 HIV-positive and 104 HIV negative participants, 63% females, median age 40.7 years (IQR 35.4 – 47.7) were included. HIV-positive individuals were older (+6 years, p <0.01) and had a higher CIMT (p <0.01). No clear patterns of inflammation were identified by use of PCA. Following ANCOVA, nine immune markers differed significantly between HIV-positive and HIV-negative participants, including PDL1. PDL1 was independently associated with CIMT, but upon stratification this effect remained for HIV-negative individuals only.

**Conclusion:**

HIV positive patients on stable ART and HIV negative controls had similar immune activation patterns. CVD risk in HIV-positive participants was mediated by inflammation markers included in this study.

## Introduction

HIV is a global public health concern with an estimated 38 million people living with HIV (PLWH) (1). Since the widespread roll-out of antiretroviral therapy (ART) life expectancy has improved substantially and age-associated non-communicable diseases (NCDs) are seen frequently in PLWH (2–4). One of the leading NCDs in HIV infection is cardiovascular disease (CVD) (5). PLWH have been shown to have an increased risk of CVD compared to the general population, and this is likely to be causally related to the triad of a direct effect of the HIV virus, metabolic side effects of ART and presence of traditional CVD risk factors (6). HIV infection results in immune activation. Although ART reduces immune activation, immune activation levels do not drop to those observed in the general population (7, 8). Many studies have attempted to identify markers of immune activation associated with an increased risk of CVD, either to understand the pathophysiology of the increase in CVD risk or to improve risk prediction. In the SMART cohort interleukin 6 (IL-6) and d-dimer were associated with an increase in CVD events (9). In multiple cross-sectional studies including HIV-positive people a wide diversity of immune markers have been related to CVD risk as assessed with surrogate markers for CVD like carotid intima-media thickness (CIMT) and coronary calcium score, with no consistent patterns when pooling the available evidence (10, 11). Recently two immune clusters were identified that could respectively identify and exclude high risk plaques in patients with suspected coronary artery disease (12). In PLWH clusters of immune markers were identified that were associated with heart failure and overall mortality (13). Also, in areas outside the CVD research field patterns of immune activation were found to be associated with disease. In patients with chronic myeloid leukaemia, for example, two distinct clusters of soluble plasma proteins were seen at diagnoses, linked to high versus low BRC-ABL expression (14, 15). In another study a cytokine signature was identified that was associated with chronic fatigue syndrome, with an area under the curve of 0.73 (15).

The aim of this paper is to assess whether patterns of immune activation can be identified that are related to HIV infection and CVD risk assessed with CIMT.

## Methods

This analysis is a sub-analysis of a cross-sectional study that has been described in detail previously (16). In brief, the cross-sectional study included HIV-positive participants not yet on ART, on first-line and on second-line ART, as well as HIV-negative controls in the inner city of Johannesburg, South Africa, from July 2016 to November 2017. A total of 197 participants on second-line ART were recruited from a randomised controlled trial (RCT) comparing low dose darunavir to the then-standard drug of second line ART, lopinavir (17). Inclusion criteria for this RCT were HIV-positivity and being on stable second-line ART for at least six months. The group of HIV-negative controls consisted of 153 participants, who were family members or friends of the HIV-positive participants (either treatment naïve, on first-line or on second-line ART), of the same age (± 5 years) and gender.

**Figure 1:**
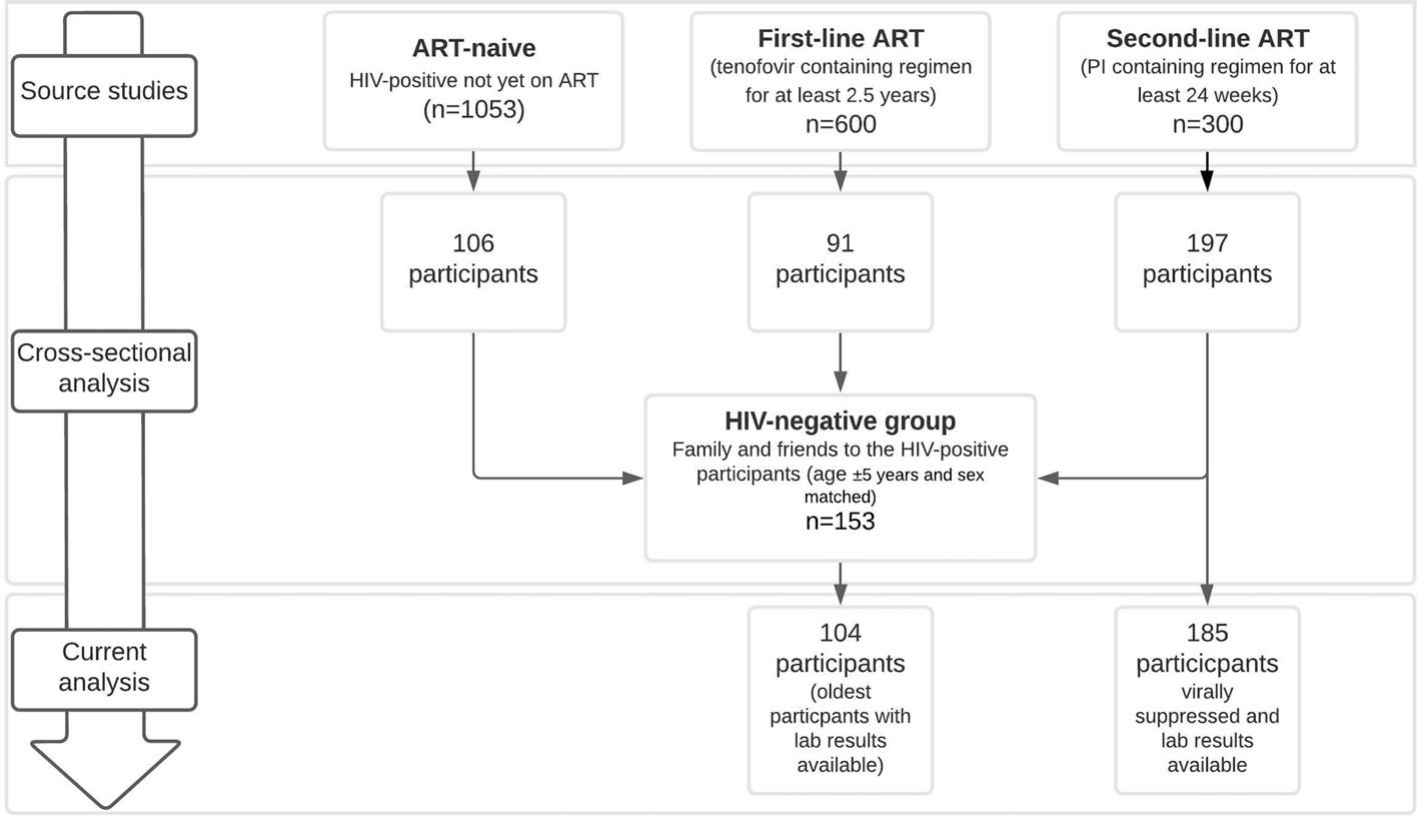
Participant selection.

In this sub-analysis all HIV-positive participants on second-line ART who were virally suppressed (viral load <50 cp/mL) with blood samples available (n=185, 93.9%) were included as well as 104 HIV-negative controls of the total of 153 controls for whom blood samples were available (**Figure 1**, Participant selection). The number of controls (n=104) was defined by available resources and the oldest controls were selected, as participants on second line therapy were on average older than the HIV negative participants. The study was approved by the Human Ethics Research Committee of the University of the Witwatersrand (ethics clearance number M160130) and performed conform the Declaration of Helsinki. All participants provided written, informed consent prior to participation.

### Data Collection

Data were collected on demographics and CVD risk factors including smoking, blood pressure, body mass index (BMI), glucose, insulin and lipids. Plasma was stored at -80 degrees Celsius. Random glucose and lipids were measured with a Cobas Integra^®^ 400 Plus analyser – hexokinase and enzymatic colorimetric methods respectively (Roche), and insulin with a Cobas E411 analyser (Roche). Hypertension was defined as use of antihypertensive drugs or a systolic blood pressure ≥140mmHg and/or a diastolic blood pressure ≥90mmHg. Diabetes as defined as the use of antidiabetic drugs or a random glucose >11.0 mmol/L.

A proximity extension assay (PEA) was applied (Olink, Uppsala, Sweden) at the UMC Utrecht (Central Diagnostic laboratory and Center for Translational Immunology, Utrecht, The Netherlands) to measure 92 immune markers simultaneously (www.olink.com) (see **Supplementary 1** for an overview of the immune markers, including the full names) (18). Protein concentrations were reported as normalized protein expression (NPX) units, which are on a Log2 scale. NPX is calculated from the Ct values from the PCR read out.

CVD risk was assessed with a CIMT measurement. A Siemens Acuson p500 ultrasound [Siemens Healthcare (Pty) Ltd, South Africa] was used and scans were obtained in B-mode with a ≥ 7.0 MHz linear probe. The near and far wall of the common carotid artery (CCA) were measured at three standardised angles at both the right and left side. Images were read off-line, in batch, using the semi-automatically Artery Measurement System software (Chalmers University, Götenburg, Sweden) and adjusted manually if needed. CIMT was reported as the mean of the mean thickness of the near and far wall across all six angles of the common carotid artery [mean CCA intima-media thickness (IMT)].

### Statistical Analysis

Descriptive data were presented as median with interquartile range for continuous variables and count with percentages for categorical outcomes. Differences in population characteristics were tested with a Mann-Whitney U test and a Chi square or Fisher’s exact test as appropriate. Data were explored from several angles using three different statistical methods. First, volcano plot analyses were performed to identify different expressed proteins between HIV negative and HIV-positive participants using Mann-Whitney U tests with Benjamini and Hoch correction for false discovery rate (FDR). Log2 fold change was calculated with NPX values on linear scale (2^^NPX^). Second, two principal component analyses (PCA) were performed to identify patterns of immune activation. The first PCA included all immune markers. The second PCA included a selection of the 30 markers with the greatest discriminatory power between HIV-positive and HIV-negative participants (19), following the methodology of Babu et al. (20). To select these 30 markers, the following steps were taken. First, Mann-Whitney U tests were conducted on the association between each marker and HIV status and ranked by p-value. Next, a random forest analysis was performed to identify markers with high discriminatory power between HIV-positive and HIV-negative study participants. This analysis resulted in two outcomes, namely Random Forest Accuracy and Random Forest Gini index. To finally select the top 30 immune markers for the second PCA, the inflammation markers that were in the top 50 in all three metrics were first selected. Second, the markers identified in the top 50 by only two metrics were sorted by ascending Mann-Whitney p-value and selected sequentially into the list of the top 30 markers until the list of 30 was complete.

Third, to further explore the data, the relation between HIV status and immune markers was tested with ANCOVA models correcting for age, sex and current smoking as smoking is strongly related to inflammation (21). To account for FDR, p-values were adjusted using the Benjamini & Hochberg FDR method (22). All immune markers with a p-value <0.1 were considered to be potentially relevant and kept in the analysis. The distribution of these immune markers was inspected with a box plot to ensure that results were not relying on outliers. A FDR p-value <0.05 was considered statistically significant. Network analysis was performed by use of the STRING – Protein-Protein Interaction Network Functional Enrichment Analysis (https://string-db.org/).

In a final analysis, immune markers with a FDR-adjusted p-value <0.1 were included in linear regression analyses to assess the role of immune activation in CVD risk as estimated by CIMT in HIV-positive participants. In the first model mean CCA-IMT was investigated as a function of HIV status, adjusted for age and sex. A second model was further adjusted for the selected immune markers, in a third model classical CVD risk factors were added (waist circumference, LDL cholesterol, systolic blood pressure, current smoking and glucose). Finally, the fully adjusted model was stratified on HIV status. Descriptive analyses were performed in SPSS Version 25.0. Armonk, NY: IBM Corp. All other analyses were performed in R software (version 3.6.3).

## Results

A total of 289 participants were included in the analysis, 185 (64%) PLWH and 104 (35%) HIV-negative participants. PLWH were older and more often female than HIV-negative participants (both p <0.01). Median time since HIV infection was 9 years and median duration on ART was 8 years. Seven (4%) participants were on ART less than two years. PLWH had higher cholesterol levels and higher intima-media thickness (**Table 1**).

**Table 1:** Participant characteristics.

	HIV-negatives (n = 104)	HIV-positives (n = 185)	*P*
Gender, female	53 (51)	129 (69.7)	0.002
Age in years	36.5 (32.3 - 46.5)	42.5 (38.1 - 48.6)	<0.001
Years since HIV diagnosis		9.0 (7.0 - 12.4)	
Years on ART		8.0 (6.0 – 10.5)	
Current smoking	38 (37.3)	17 (9.2)	<0.001
BMI (kg/m2)	25.3 (21.5 - 30.9)	26.5 (23.1 - 32.0)	0.120
Waist to hip ratio (cm) (n=284)	0.86 (0.82 - 0.90)	0.87 (0.83 - 0.91)	0.047
Systolic blood pressure (mmHg)	122.5 (116.1 - 133.6)	120.0 (111.0 - 131.5)	0.036
Diastolic blood pressure (mmHg)	78.3 (72.5 - 84.8)	78.5 (70.3 - 83.5)	0.295
Hypertension^#^	27 (26.0)	41 (22.2)	0.465
Total cholesterol (mmol/L) (n=281)	4.34 (3.75 - 4.77)	4.70 (4.18 - 5.35)	<0.001
HDL cholesterol (mmol/L) (n=281)	1.26 (1.09 - 1.68)	1.34 (1.12 - 1.58)	0.816
LDL cholesterol (mmol/L) (n=281)	2.26 (1.73 - 2.77)	3.01 (2.40 - 3.61)	<0.001
Triglycerides (mmol/L) (n=281)	1.06 (0.82 - 1.34)	1.26 (0.90 - 1.71)	0.002
Random glucose (mmol/L) (n=278)	4.8 (4.4 - 5.3)	4.6 (4.4 - 5.0)	0.011
Insulin (mE/L) (n=284)	9.3 (4.4 - 21.0)	14.0 (7.4 – 27.0)	0.003
Diabetes (n=278)*	0 (0.0)	5 (2.9)	0.161
CD4 cell count (cells/mm3) (n=177)		623 (430 - 804)	
Max CCA-IMT	0.618 (0.549 - 0.700)	0.668 (0.602 - 0.757)	<0.001
Mean CCA-IMT	0.540 (0.490 - 0.607)	0.566 (0.518 - 0.655)	0.003
Max bulb-IMT	0.736 (0.621 - 0.895)	0.813 (0.700 - 0.976)	0.010

Continuous outcomes were presented as median with interquartile range; categorical data as count with percentages.

^#^defined as use of antihypertensive drugs or a systolic blood pressure ≥140mmHg and/or a diastolic blood pressure ≥90mmHg.

*defined as use of antidiabetic drugs or a random glucose >11.0 mmol/L ART, anti-retroviral therapy; BM, body mass index; CCA, common carotid artery; HDL, high density lipoprotein; IMT, intima-media thickness; LDL, low density lipoprotein.

**Figure 2:**
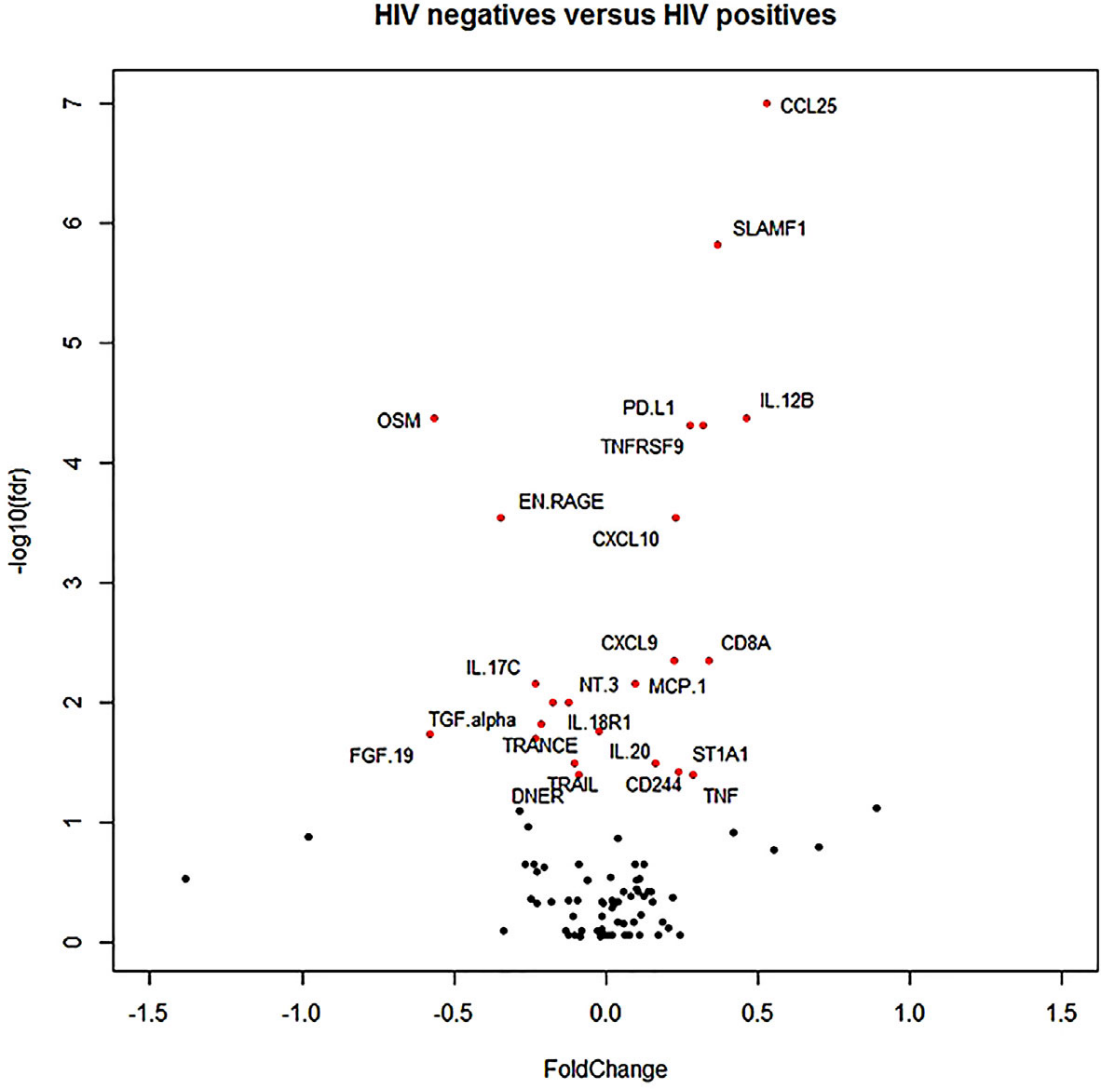
Volcano plot. The red dots indicate the markers that differed at a false discovery rate corrected p-value <0.05.

In a Volcano plot, 21 proteins differed at an FDR p <0.05 between HIV-positive and HIV-negative participants. Fold change (magnitude of change expressed in log2) was relatively low with only three markers exceeding 0.5 (where a value of 1 means a doubling of protein concentration), namely CCL25, OSM and FGF19 (**Figure 2**, Volcano plot).

PCA did not show clear patterns of inflammation markers between the HIV-negative and HIV-positive participants. In the first PCA including all immune markers the first 2 components captured 30% of the variation, and this hardly improved in a second PCA including the top 30 most discriminatory markers, where the first 2 components captured 32% of the variation (**Supplementary 2**).

Following ANCOVA, 15 immune markers were found to differ between HIV-positive and HIV-negative individuals at an FDR-adjusted p-value of <0.10, of which nine had a FDR-adjusted p-value <0.05 (**Table 2**). The top three markers included CCL25 and OSM, markers that were also identified in the volcano plot analysis. For an overview of the distribution of the 15 immune markers with a FDR adjusted p-value <0.1 see **Supplementary 3**. For an overview of the outcome of all individual immune markers see **Supplementary 4**.

**Table 2:** Immune markers in relation to HIV status.

Immune marker	Effect size of HIV status^#^	P*
CCL25	0.54788	<0.001
SLAMF1	0.35499	<0.001
OSM	-0.59971	<0.001
PDL1	0.26113	<0.001
IL18R1	-0.26237	0.002
CCL28	-0.24386	0.025
TRANCE	-0.24402	0.046
CXCL10	0.36137	0.046
TNFRSF9	0.18390	0.046
FGF21	-0.51678	0.054
FGF19	-0.39087	0.055
CD8A	0.28601	0.058
IL13	-0.22815	0.063
EN-RAGE	-0.29137	0.063
LAPTGFbeta1	-0.15852	0.070

^#^values adjusted for age, sex and current smoking, *adjusted using the Benjamini & Hochberg false discovery rate.

**Figure 3:**
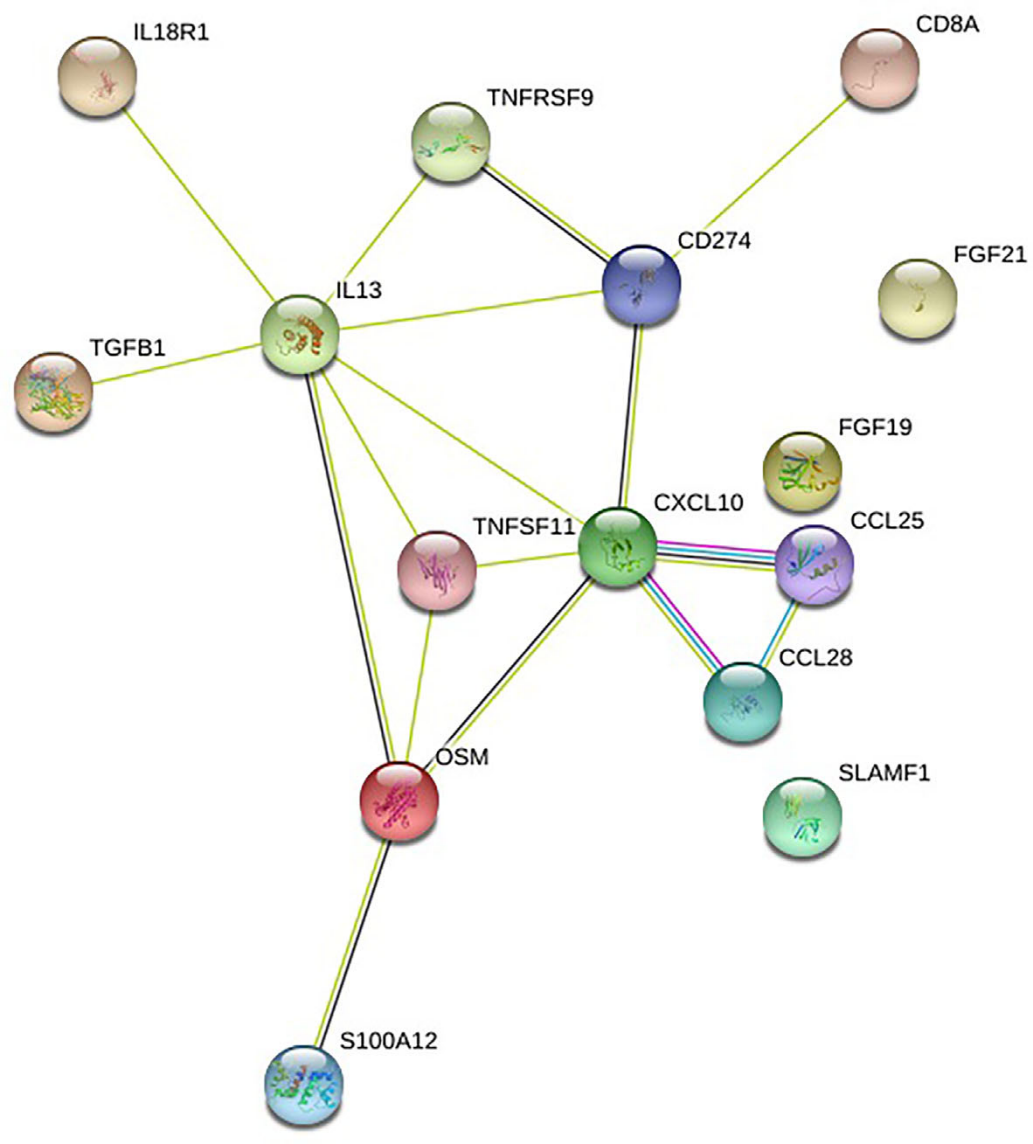
Network of immune markers. Source: https://string-db.org/. Legend: CD274 is PDL1, TNFSF11 is TRANCE, S100A12 is EN-RAGE. See **Appendix 1** for the abbreviations. Meaning of lines: blue: from curated databases, pink: experimentally determined, green: gene neighborhood, red: gene fusions, blue: gene co-occurrence, light green: text mining, black: co-expression.

These 15 immune markers identified by the ANCOVA analysis are related to one another in networks (https://string-db.org/) (**Figure 3**).

### Immune Activation and CVD Risk

Age was related to CIMT in the first model. Following additional adjustment for all 15 immune markers (**Table 2**), age (β=0.008, p <0.001) and PDL1 (β=0.037, p=0.004) were associated with CIMT. Further adjustment for CVD risk factors did not change these findings. Systolic blood pressure was significantly associated with CIMT; LDL cholesterol non-significantly. In fully adjusted models stratified on HIV status, the association of PDL1 with CIMT was attenuated (β=0.030, p=0.09) in HIV-infected participants, whereas the association remained significant for HIV-negative participants (β =0.057, p=0.02). Systolic blood pressure remained significantly associated with CIMT, β=0.001, p=0.02) for the HIV-negative participants (**Table 3**).

**Table 3:** HIV, immune activation and carotid intima-media thickness.

		β-coefficient	SE	*p*
Model 1	HIV	0.002	0.010	0.858
	Age	0.007	0.001	<0.001
	Male sex	0.001	0.010	0.949
Model 2^#^	HIV	-0.006	0.014	0.693
	Age	0.008	0.001	<0.001
	Male sex	-0.010	0.012	0.413
	PDL1	0.037	0.013	0.004
Model 3^&^	HIV	-0.009	0.015	0.532
	Age	0.007	0.001	<0.001
	Male sex	-0.011	0.014	0.433
	PDL1	0.039	0.013	0.003
	LDL-C	0.012	0.006	0.079
	SBP	0.001	0.003	0.036
Model 3 – HIV-positives only^&^	Age	0.007	0.001	<0.001
	Male sex	-0.007	0.017	0.677
	PDL1	0.030	0.017	0.085
	LDL-C	0.015	0.008	0.055
	SBP	<0.001	<0.001	0.330
Model 3 – HIV-negatives only^&^	Age	0.008	0.001	<0.001
	Male sex	-0.031	0.028	0.266
	PDL1	0.057	0.023	0.017
	LDL-C	0.004	0.012	0.754
	SBP	0.001	<0.001	0.023

Model 1: HIV, age and sex. Model 2: HIV, age, sex, and all immune markers included in **Table 2**. Model 3: HIV, age, sex, all immune markers included in **Table 2**, waist circumference, LDL cholesterol, systolic blood pressure, current smoking and glucose ^#^Immune markers with p>0.10 not included in the table. ^&^Immune markers and CVD risk factors with p>0.10 not included in the table.

## Discussion

In this paper, the largest so far on proteomics in the field of HIV research, we evaluated immune markers in people with HIV who were virally suppressed on ART and participants without HIV. We could not identify distinct immune patterns between the groups. Nine immune markers were significantly different between the groups on an individual level, of which PDL1 was also related to CVD risk, independent of CVD risk factors.

Our paper adds to the growing body of literature on proteomics in HIV infection. Recently four papers were published describing the results of the Olink panel inflammation while comparing HIV-positive to HIV-negative individuals. Babu et al. (20) included 53 PLWH on stable ART and 41 HIV-negative participants. In line with our findings, SLAMF1 levels were significantly higher, and TRANCE levels significantly lower in PLWH on ART as compared to controls. However, the other seven immune markers that we found to differ at a p <0.05 level were not confirmed in their analysis. We found that CD8A levels were increased in PLWH, although just not statistically significant (p=0.058). Babu et al. reported this value to be significantly increased in PLWH on ART compared to controls. Besides, they found eight other markers to differ significantly between PLWH on ART and HIV-negative controls (NT3, CD5, TRAIL, ADA, MMP1, CST5, 4E-BP1, CCL23). In a second paper by Babu et al. 22 PLWH on stable ART and 22 HIV-negative controls were included (23). Only three immune markers were found to differ significantly between the groups, including TRANCE (lower in PLWH). DeFilippi et al. included 89 PLWH on stable ART and 46 HIV-negative controls. Results cannot be compared directly due to differences in statistical approach. They found amongst others, CCL25, TRANCE and CD8A to differ significantly between the groups (p <0.05 in a volcano plot) (24). The findings in this study are largely in line with our study as they also found that the magnitude of difference in immune markers between the groups was rather low (fold change predominantly between -0.4 and +0.4). DeFillippi et al. included three other Olink panels next to the Inflammation panel. Despite the vast number of immune markers, following PCA analysis 46% of the variation in the data could be explained. We found that when using the inflammation panel, 32% of the variation could be explained. This indicates that the studied immune markers were only moderately capable to capture the extent of the immune response, and that adding extra immune markers resulted in a slight increase of explained variance. Lemma et al. (25) included 43 HIV-positive and 43-HIV negative children with a median age of 60 months from Ethiopia. They found in line with our findings, levels of OSM and TRANCE to be significantly lower in HIV-positive participants. On the contrary, they found that FGF21 levels were significantly higher in HIV-positive children, while we found borderline significant lower values in PLWH (p =0.054). Besides, they found seven other immune markers to differ significantly between PLWH and HIV-negative controls.

Considering all the findings so far there is still a marked heterogeneity and there is no consistent immune pattern when comparing the studies. This may be due to differences in ethnic background, age and comorbid conditions like smoking, overweight and hypertension.

However, there is one consistent finding across all studies, namely a lower level of TRANCE in PLWH as compared to HIV-negative controls. TRANCE, also known as RANKL, OPF, OPGL or TNFSF11, has several biological functions. It acts as a survival factor for dendritic cells and is important for immune tolerance. It is involved in the development of tissues like lymphoid tissue but is mainly known for its important role in bone homeostasis through osteoclast activation and differentiation (26).

Osteopenia is commonly seen in HIV infection and PLWH are at increased risk of fractures (27–29).

Osteoclast activation is regulated through the TRANCE/osteoprotegrin (OPG) axis, where OPG inactivates TRANCE and hence stops osteoclast activation and profileration (30).

PLWH were found to have a higher percentage of TRANCE expressing B-cells and a lower percentage of OPG-expressing B-cells compared to HIV negative controls. However, plasma levels of TRANCE and OPG were not related to osteopenia and OPG levels were found to be higher in PLWH compared to HIV-negative controles (30). OPG levels were not found to differ between PLWH and HIV-negative controls in our study. A possible explanation for the observation of lower soluble TRANCE levels in PLWH might be that TRANCE is produced in several tissues outside the bone (31) and as such it might not reflect bone homeostasis accurately.

CCL25 is the immune marker that differed the most between PLWH and HIV-negative controls in our study. It is a chemokine with anti-apoptotic activity and has CCL9 as its ligand (32). In an SIV/macaque model CCL25 mRNA was downregulated in lymphoid tissue, and this was associated with an increase in apoptotic cells and proliferation of cells in lymph nodes (33). Besides, CCL25 is expressed in the small intestine and known to play a major role in trafficking of CCL9+ CD4+ T-cells to the gut, needed for maintaining or restoration of gut immunity. CCL25 mRNA in the small intestine was found to be decreased by five times in PLHV compared to healthy controls. This was linked to failing gut immunity, decreased numbers of CCR9+ CD4+ T-cells in the gut, while the proportion of these cells was increased in the peripheral blood (34). The increased plasma level of CCL25 in PLHV in this analysis warrants further research as it might provide new insights in the altered homing of CCR9+ CD4+ cells to the gut in HIV-positive individuals, even after initiation of ART.

SLAMF1, also known as CD150, is an Ig-like receptor and a costimulatory molecule found in a variety of immune cells. It can induce release of interferon-γ by T helper type 1 cells, which is a potent cytokine in the immune response to viruses (35, 36). Babu et al. also found this immune marker to be increased in PLWH compared to controls (20).

OSM (oncostatin-M) has the strongest effect in differentiating between PLWH and HIV-negative controls in our study, with lower values for PLWH. Lemma et al. (25) had the same finding in children. OSM is part of the IL-6 family and is active in multiple cell types where it influences cell differentiation and inflammation (37). The OSM gene was found to be overexpresses in chronic, untreated HIV infection (38). Increased levels of OSM have been related to neurodegenerative diseases like multiple sclerosis and HIV associated neuro-cognitive disorder (39). The meaning of the lower level found in our study has yet to be determined.

Another finding was the lower levels of circulating IL18-receptor 1 (IL18R). This is an established receptor on Th1 lymphocytes and responsible for the binding of IL18. IL-18 is a pro-inflammatory cytokine that is mostly known for stimulation of interferon-ϒ production by Th1 cells (40). The soluble form of IL18R1 (also called sIL-18Ralpha) is not able to block IL-18 in plasma, unless the second part of the receptor, sIL-18Rbeta is present and both soluble receptors are linked to the Fc domain of IgG1 (41). In our study the level of IL-18 nor IFN-gamma differed between the groups. The meaning of the lower levels of the IL18R1 has to be established in further research.

Finally, it is worth reflecting on the role of programmed death ligand 1 (PDL-1) in HIV infection as plasma levels were increased in PLWH compared to HIV-negative controls. Besides, it was associated with CIMT independent of classic CVD risk factors. PDL1, also known as CD274, is a marker of lymphocyte activation and is expressed on both CD4+ T-cells and B-cells (42). PDL-1 and its substrate, PD-1, were found to be increased on CD4+ and CD8+ T-cells and B-cells in HIV-positive individuals (43). Blocking of the PD-1/PDL-1 axis in SIV infected monkeys led to an expansion of virus-specific CD8+ T-cells and a reduction in viral load (42). PDL-1 is a marker associated to T-cell exhaustion. We also found that an increase in PDL-1 was related to CIMT independent of CVD risk factors. Activation of the PDL-1/PD-1 axis has been associated with chronic inflammation in the atherosclerotic plaque, resulting in the influx of inflammatory T-cells and loss of tissue-protective T-cell immunity (44). Upon stratification on HIV status PDL-1 was no longer significant in the HIV-positive group while it became more pronounced in the HIV-negative group. LDL-C became borderline significant in the HIV-positive group, while the already non-significant relationship with CIMT was further weakened in the HIV-negative group. This suggests that pathophysiology of plaque development differs between PLWH and HIV-negative controls, as has been described before (45).

Our study has several strengths. It is the largest study so far on proteomics comparing HIV-positive to HIV-negative participants. The HIV-positive group was a homogeneous group as all people were virally suppressed on stable ART. Participants represent a population with the highest HIV burden globally and the majority of PLWH in our study were women. Participants were well characterized including CVD risk assessment. There are also some limitations to be mentioned. Although the HIV-negative group was recruited from the same background as the PLWH, demographics like age and gender differed between the groups. Although we corrected for this, there might have been unmeasured confounding influencing the comparison between the groups.

We did not have information on nadir CD4 count and although the majority of people were on ART for several years, there was a small proportion on ART for a relatively short period. Studying better-defined subgroups with comparable nadir CD4 cell counts or duration of treatment might have revealed more pronounced immune patterns. For CVD risk we used a surrogate outcome. Although an increase in CIMT is closely linked to an increase in CVD risk, it is an intermediate outcome. Finally, our population was still relatively young which implies a low CVD risk.

In summary, no clear pattern of immune activation was found that was able to differentiate between PLWH on stable ART and HIV-negative controls. Analysis of individual immune markers showed nine markers that differed significantly between the groups, some with a known role in HIV pathogenesis. PDL1 was related to CVD risk as assessed with CIMT, but this effect was only seen in the HIV-negative group. A way to take the search for immune patterns forward would be to combine the results of existing datasets including the Olink inflammation panel in HIV-positive and HIV-negative individuals. This would increase the power of data reduction methods, like a PCA, to identify immune patterns. Further research should include well-defined groups of HIV-positive patients, defining groups based on nadir CD4-cell count, time since HIV infection, time on ART and time with adequate viral suppression on ART. Besides, it would be worthwhile to repeat the measurements in older patients as CVD risk becomes more pronounced with increasing age. The best insights would be gained by a longitudinal study were blood samples preceding a CVD events could be analysed and compared to blood samples of participants without CVD event.

## Data Availability Statement

The raw, deidentified data supporting the conclusion of this article will be made available by the author, without undue reservation.

## Ethics Statement

The studies involving human participants were reviewed and approved by Human Ethics Research Committee of the University of the Witwatersrand (ethics clearance number M160130). The patients/participants provided their written informed consent to participate in this study.

## Author Contributions

AV, SN, CS, and WV conceived of the presented idea. AV, KK-G, and WV collected the data. SN and ED supervised the laboratory measurements. AV, CD, and ED analyzed the data. AV wrote the manuscript. ED and SN helped with the interpretation of the findings. All authors contributed to the article and approved the submitted version.

## Funding

The laboratory analysis and the reading of the CIMT images was sponsored by South Africa Medical Research Council, as an addition to grand named ‘*Development of a better & more robust second-line antiretroviral regimen for HIV infection*’.

## In Memoriam

This work is dedicated to the memory of C. Serenata, 1969 - 2021.

## Conflict of Interest

The authors declare that the research was conducted in the absence of any commercial or financial relationships that could be construed as a potential conflict of interest.
