# Supplementary material for: Patterns of Immune Activation in HIV and Non HIV Subjects and Its Relation to Cardiovascular Disease Risk
Source: Front Immunol. 2021 Jul 5;12:647805. doi: 10.3389/fimmu.2021.647805 (PMC8287326; doi:10.3389/fimmu.2021.647805)
Supplement: Supplementary file 1 [file DataSheet_1.pdf]

Overview	Inflammation markers included in the Olink ‘inflammation’ panel
4E-BP1	Eukaryotic translation initiation factor 4E-binding protein 1
ADA	Adenosine Deaminase
ARTN	Artemin
AXIN1	Axin-1
Beta-NGF	Beta-nerve growth factor
CASP8	Caspase-8
CCL3	C-C motif chemokine 3
CCL4	C-C motif chemokine 4
CCL11	Eotaxin
CCL19	C-C motif chemokine 19
CCL20	C-C motif chemokine 20
CCL23	C-C motif chemokine 23
CCL25	C-C motif chemokine 25
CCL28	C-C motif chemokine 28
CD244	Natural killer cell receptor 2B4
CD40	CD40L receptor
CD5	T-cell surface glyocprotein
CD6	T-cell surface glycoprotein CD6 isoform
CD8A	T-cell surface glycoprotein CD8 alpha chain
CDCP1	CUB domain-containing protein 1
CSF1	Macrophage colony-stimulating factor 1
CST5	Cystatin D
CX3CL1	Fractalkine
CXCL1	C-X-C motif chemokine 1
CXCL5	C-X-C motif chemokine 5
CXCL6	C-X-C motif chemokine 6
CXCL9	C-X-C motif chemokine 9
CXCL10	C-X-C motif chemokine 10
CXCL11	C-X-C motif chemokine 11
DNER	Delta and Notch-like epidermal growth factor-related receptor
EN-RAGE	Protein S100-A12
FGF19	Fibroblast growth factor 19
FGF21	Fibroblast growth factor 21
FGF23	Fibroblast growth factor 23
FGF5	Fibroblast growth factor 5
Flt3L	Fms-related tyrosine kinase 3 ligand
GDNF	Glial cell line-derived neutrotrophic factor
HGF	Hepatocyte growth factor
IFN-gamma	Interferon gamma
IL1alpha	Interleukin-1 alpha
IL2	Interleukin-2
IL2RB	Interleukin-2 receptor subunit beta
IL4	Interleukin-4
IL5	Interleukin-5
IL6	Interleukin-6
IL7	Interleukin-7
IL8	Interleukin-8

IL10	Interleukin-10
IL10RA	Interleukin-10 receptor subunit alpha
IL10RB	Interleukin-10 receptor subunit beta
IL12B	Interleukin-12 subunit beta
IL13	Interleukin 13
IL15RA	Interleukin-15 receptor subunit afpha
IL17A	Interleukin 17A
IL17C	Interleukin 17C
IL18	Interleukin 18
IL18R1	Interleukin-18 receptor 1
IL20	Interleukin-20
IL20RA	Interleukin-20 receptor subunit alpha
IL22RA1	Interleukin-22 receptor subunit alpha-1
IL24	Interleukin-24
IL33	Interleukin-33
LAPTGFbeta1	Latency-associated peptide transforming growth factor beta-1
LIF	Leukemia inhibitory factor
LIFR	leukemia inhibitory factor receptor
MCP1	Monocyte chemotactic protein-1
MCP2	Monocyte chemotactic protein-2
MCP3	Monocyte chemotactic protein-3
MCP4	Monocyte chemotactic protein-4
MMP1	Matrix metalloproteinase-1
MMP10	Matrix metalloproteinase-10
NRTN	Neurturin
NT3	Neutrotrophin-3
OPG	Osteoprotegerin
OSM	Oncostatin-M
PDL1	Programmed cell death 1 ligand 1
SCF	Stem cell factor
SIRT2	SIR2-like protein
SLAMF1	Signaling lymphocyte activation molecule family 1
ST1A1	Sulfotransferase 1A1
STAMBP	STAM-binding protein
TGF-alpha	Transforming growth factor alpha
TNF	tumor necrosis factor
TNFB	tumor necrosis factor-beta
TNFRSF9	tumor necrosis factor receptor superfamily member 9
TNFSF14	tumor necrosis factor ligand superfamily member 14
TRAIL	TNF-related apoptosis-induced ligand
TRANCE	TNF-related activation-induced cytokine
TSLP	Thymic stromal lymphopoietin
TWEAK	Tumor necrosis factor (Ligand) superfamily member 12
uPA	Urokinase-type plasminogen activator
VEGF-A	Vascular endothelial growth factor A
