# Supplementary material for: Patterns of Immune Activation in HIV and Non HIV Subjects and Its Relation to Cardiovascular Disease Risk
Source: Front Immunol. 2021 Jul 5;12:647805. doi: 10.3389/fimmu.2021.647805 (PMC8287326; doi:10.3389/fimmu.2021.647805)
Supplement: Supplementary file 2 [file DataSheet_2.pdf]

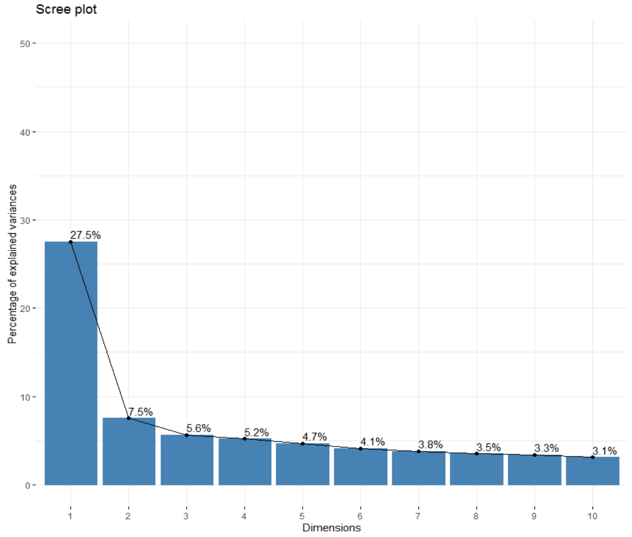

Fig A. Scree plot of a PCA including all immune markers.

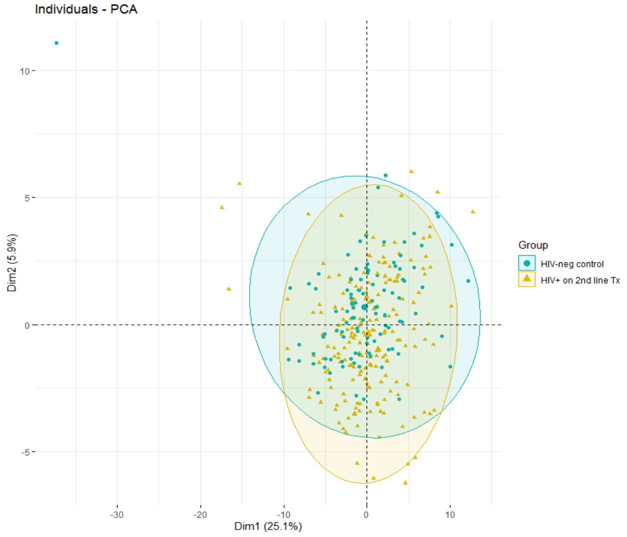

Fig B. PCA including all immune markers. Dimensions 1 & 2 according to HIV status**.**

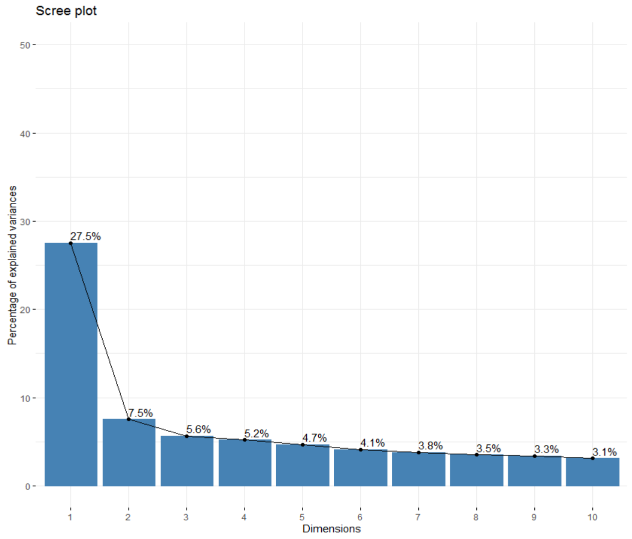

Fig C. Scree plot of a PCA including the top 30 immune markers with the most discriminatory power.

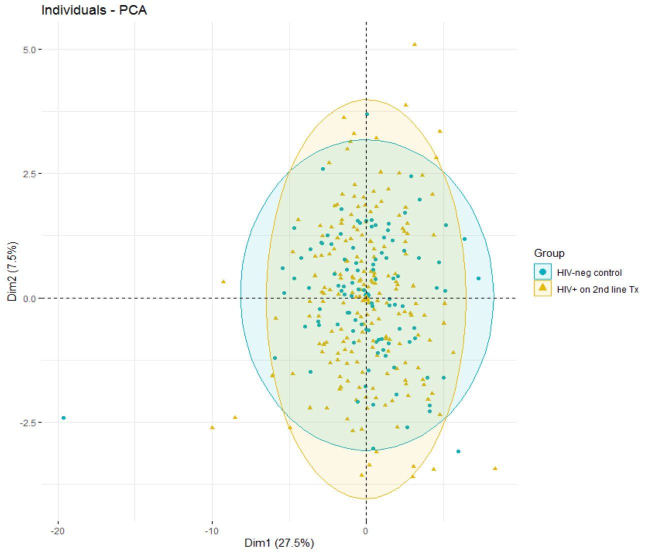

Fig D. PCA including the top 30 immune markers with the most discriminatory power. Dimensions 1 & 2 according to HIV status.

Appendix 2. Figure A-D. Principal component analysis by HIV status.
PCA; principal component analyis, Tx; treatment.
