# Supplementary figures and images for: Patterns of Immune Activation in HIV and Non HIV Subjects and Its Relation to Cardiovascular Disease Risk
Source: Front Immunol. 2021 Jul 5;12:647805. doi: 10.3389/fimmu.2021.647805 (PMC8287326; doi:10.3389/fimmu.2021.647805)

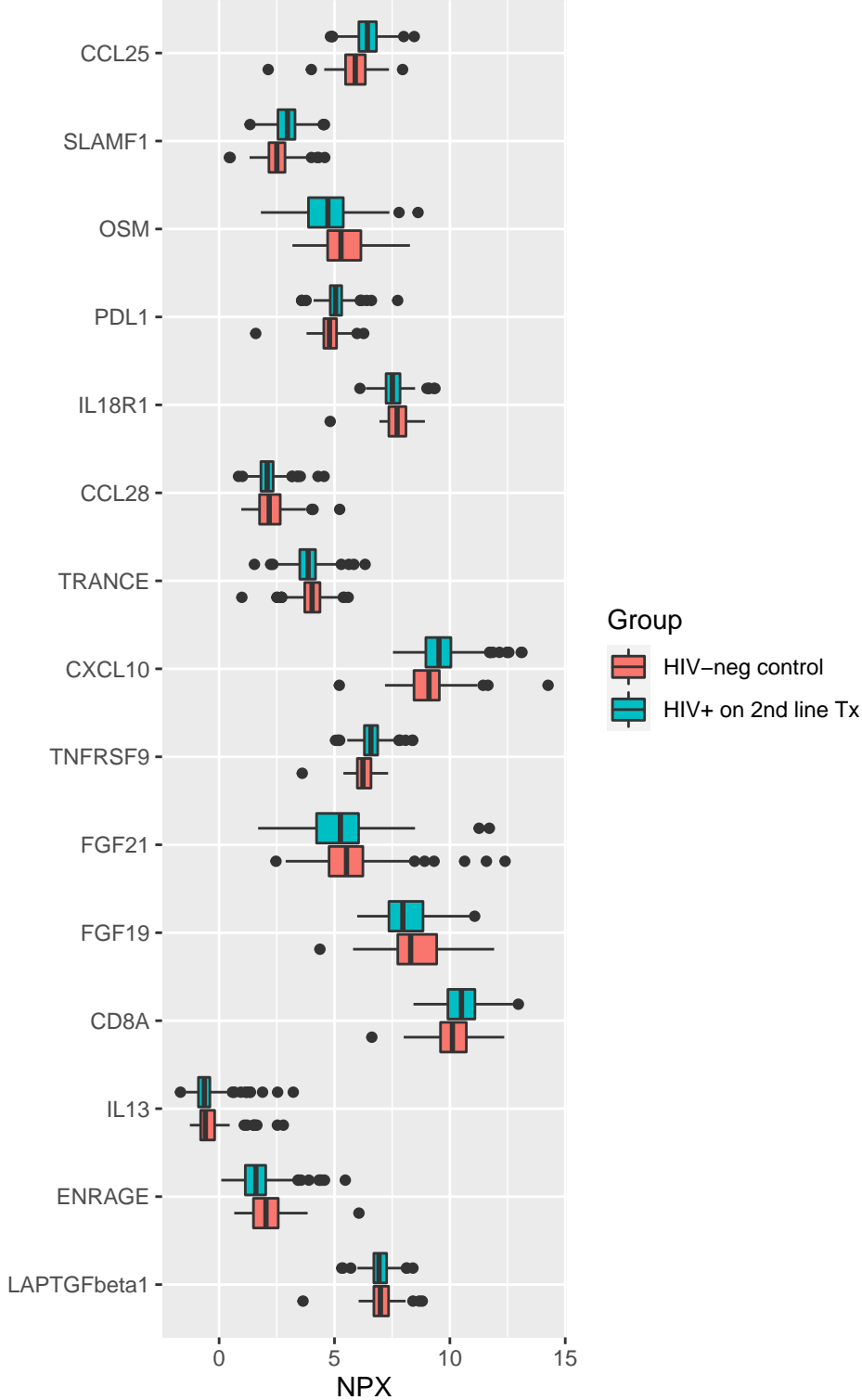

Supplement: Supplementary file 3 [file DataSheet_3.pdf]
