# Supplementary material for: Patterns of Immune Activation in HIV and Non HIV Subjects and Its Relation to Cardiovascular Disease Risk
Source: Front Immunol. 2021 Jul 5;12:647805. doi: 10.3389/fimmu.2021.647805 (PMC8287326; doi:10.3389/fimmu.2021.647805)
Supplement: Supplementary file 4 [file DataSheet_4.pdf]

Supplementary table. Immune markers in relation to HIV status.

Immune markers	*P*
CCL25	<0.0001
SLAMF1	0.0006
OSM	0.0006
PDL1	0.0006
IL18R1	0.0016
CCL28	0.0247
TRANCE	0.0461
CXCL10	0.0461
TNFRSF9	0.0461
FGF21	0.0545
FGF19	0.0547
CD8A	0.0577
IL13	0.0629
EN-RAGE	0.0629
LAPTGFbeta1	0.0703
TGF-alpha	0.1032
IL17C	0.1128
TNF	0.1425
CXCL9	0.1706
MCP1	0.1981
TNFSF14	0.1981
IL12B	0.1981
4E-BP1	0.1981
ST1A1	0.1981
IL20	0.2121
SCF	0.2265
VEGFA	0.2465
IL18	0.2465
NT3	0.2729
IL8	0.2979
CCL11	0.3007
AXIN1	0.3046
TRAIL	0.3331
DNER	0.3331
IL1alpha	0.3678
CD244	0.3819
CXCL1	0.3819
FGF23	0.3827
CD5	0.3827
CASP8	0.3827
TSLP	0.4947
CCL23	0.4947
CCL4	0.5113
MMP1	0.5113
IL24	0.5113
CX3CL1	0.5201
Continued	
MCP2	0.5782
IL7	0.6833
IL5	0.6833
CXCL11	0.6953
CCL20	0.7122
IL2	0.7234
IL10RA	0.7724
MCP4	0.7868
IL6	0.8461
CSF1	0.8482
CXCL6	0.8583
ARTN	0.8645
CD6	0.8652
STAMBP	0.8652
IL2RB	0.8727
TNFB	0.9010
MCP3	0.9052
IL15RA	0.9052
IL22RA1	0.9052
CXCL5	0.9052
IL10	0.9052
Flt3L	0.9052
CD40	0.9052
IFN-gamma	0.9052
NRTN	0.9052
TWEAK	0.9052
OPG	0.9084
IL17A	0.9271
Beta-NGF	0.9295
CDCP1	0.9451
IL20RA	0.9451
IL33	0.9451
LIF	0.9451
ADA	0.9451
FGF5	0.9471
IL4	0.9471
uPA	0.9489
CST5	0.9489
LIFR	0.9489
CCL19	0.9489
IL10RB	0.9489
HGF	0.9489
MMP10	0.9489
SIRT2	0.9489
CCL3	0.9557
GDNF	0.9795

values adjusted for age, sex and current smoking, *adjusted using the Benjamini & Hochberg false discovery rate
